# Evolution of Polyphenolic Compounds and Sensory Properties of Wines Obtained from Grenache Grapes Treated by Pulsed Electric Fields during Aging in Bottles and in Oak Barrels

**DOI:** 10.3390/foods9050542

**Published:** 2020-04-30

**Authors:** Marcos Andrés Maza, Juan Manuel Martínez, Guillermo Cebrián, Ana Cristina Sánchez-Gimeno, Alejandra Camargo, Ignacio Álvarez, Javier Raso

**Affiliations:** 1 Departamento de Ciencias Enológicas y Agroalimentarias, Facultad de Ciencias Agrarias, Universidad Nacional de Cuyo, M5528AHB Mendoza, Argentina; mmaza@fca.uncu.edu.ar (M.A.M.); acamargo@fca.uncu.edu.ar (A.C.); 2 Tecnología de los Alimentos, Facultad de Veterinaria, Instituto Agroalimentario de Aragón-IA2, (Universidad de Zaragoza-CITA), c/Miguel Servet, 177, 50013 Zaragoza, Spain; jmmr@unizar.es (J.M.M.); guiceb@unizar.es (G.C.); anacris@unizar.es (A.C.S.-G.); ialvalan@unizar.es (I.A.)

**Keywords:** maceration-fermentation, polyphenol extraction, PEF, Grenache, sensory analysis

## Abstract

The evolution of polyphenolic compounds and sensory properties of wines obtained from *Grenache* grapes, either untreated or treated with pulsed electric fields (PEF), in the course of bottle aging, as well as during oak aging followed by bottle aging, were compared. Immediately prior to aging in bottles or in barrels, enological parameters that depend on phenolic extraction during skin maceration were higher when grapes had been treated with PEF. In terms of color intensity, phenolic families, and individual phenols, the wine obtained with grapes treated by PEF followed an evolution similar to untreated control wine in the course of aging. Sensory analysis revealed that the application of a PEF treatment resulted in wines that are sensorially different: panelists preferred wines obtained from grapes treated with PEF. Physicochemical and sensory analyses showed that grapes treated with PEF are suitable for obtaining wines that require aging in bottles or in oak barrels.

## 1. Introduction

Maceration-fermentation is the most critical stage in the red winemaking process. Phenolic composition, aroma, flavor, and aging capacity of wine largely depend on the extraction of specific compounds from the grape skins in this stage [1].

Improving the degree of extraction of polyphenols during the maceration-fermentation stage of red winemaking has been one of the most widely investigated applications of pulsed electric fields (PEF) in recent years. Several research groups working with different grape varieties have demonstrated that a PEF treatment applied to grapes before the maceration-fermentation stage allows for a reduction of the contact time of grape skins with the fermenting must, or helps to obtain wines with higher polyphenolic content [2,3,4,5,6]. This effect is attributed to a phenomenon called electroporation, which involves an alteration of cell membrane permeability as a consequence of the application of an external electric field [7].

Although certain polyphenolic compounds are located in the cell wall of grape skins and seeds, the majority of phenolic compounds responsible for the color and sensory properties of red wine are located inside the vacuoles of the cytoplasm of grape skin cells [8]. Electroporation of the cell membrane therefore improves the mass transfer of those compounds to the fermenting must during maceration. This effect can help to obtain wines with a higher concentration of polyphenols, or it can allow for an earlier removal of the grape skins from the fermentation tanks. Electroporation can thereby help reduce the demand for expensive fermentation tanks, and it can lead to savings in labor costs associated with the maceration-fermentation process.

PEF treatment has proven beneficial in obtaining fresh fermented red wine which, however, is not ready for consumption. After the maceration-fermentation stage, an aging period in bottle or in oak barrels is required. In this stage, polyphenols participate in subsequent reactions, which are those that actually exert the greatest influence on the overall sensory quality of finished wine [9]. These modifications are a consequence of precipitations or degradation-polymerization and co-pigmentation reactions that lead to the formation of new stable compounds, and which thereby bring about important changes in the sensory properties of wine [10]. Since these reactions depend to a great extent on the type and concentration of polyphenols obtained in the course of the maceration-fermentation stage, it is important to be able to gain more precise knowledge regarding the evolution of wines obtained from grapes treated with PEF during aging.

The objective of this study is to compare the evolution and sensory properties of wines obtained from untreated and PEF-treated *Grenache* grapes during bottle aging, as well as during oak aging followed by bottle aging.

## 2. Material and Methods

### 2.1. Samples and Vinification

Ca. 12,000 kg of *Grenache* grapes *(Vitis vinifera L*.) cultivated in the “Campo de Borja” appellation of origin (A.O.) located in the Spanish region of Aragón were harvested in October 2016. Brix, pH and total acidity of the grape must were 27.9 ± 0.2, 3.4 ± 0.3, and 4.9 ± 0.2 g.L^−1^ respectively.

**Figure 1:**
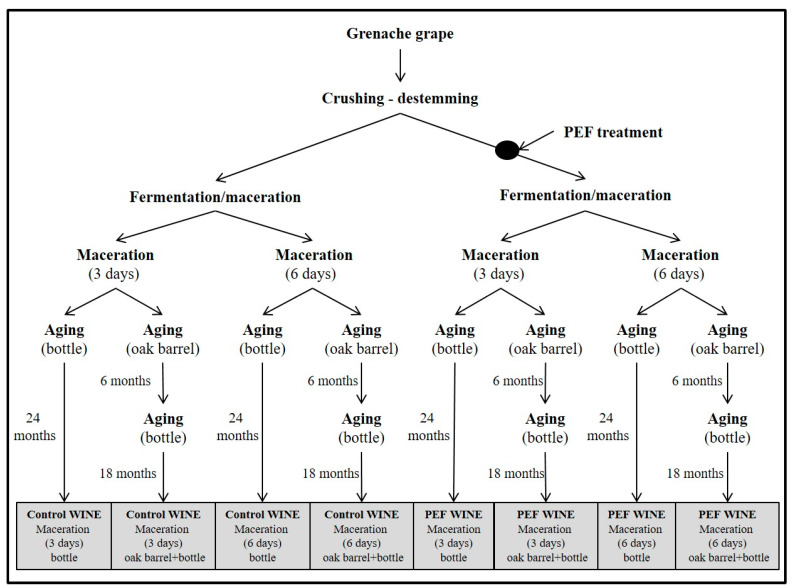
Schematic diagram of vinification and aging.

The winemaking process is shown in Figure 1. Grape mass was distributed in four fermentation tanks of 5000 L capacity each. Two of the tanks contained ca. 3000 kg of untreated grapes, and the two other tanks contained ca. 3000 kg of PEF-treated grapes.

Grape skins were removed from tanks containing untreated and PEF-treated grapes after three and six days of maceration, and fermentation was extended for 10 days.

At the end of fermentation, 100 mg of K_2_S_2_O_5_ L^−1^ were added, and wine was kept at 4 °C for a stabilization period of three months. The wine was subsequently separated into two portions. One portion was racked and aged in bottles for 24 months in a conditioned room maintained at 18 ± 1 °C. The other portion was racked and aged in new medium-toast American Oak barrels of 16 L capacity (Tonelería los Pinos, Cordoba, Spain) for six months, then racked again, bottled, and stored in bottles for a further 18 months.

### 2.2. PEF Equipment

PEF treatments were applied with a PM1-10 generator, (supplied by Energy Pulse Systems LDA, Lisbon, Portugal). A high-voltage probe (Tektronix, P6015A, Wilsonville, OR, USA) and an oscilloscope (Tektronix, TBS 1102B-EDU) were used to record and measure the shape and intensity of the pulse. The PEF generator was connected to a collinear treatment chamber with two treatment zones (2.5 cm length and 3.5 cm diameter).

The grape mass was pushed through the treatment chamber using a peristaltic pump (Rotho MS1, Ragazzini, Faenza, Italy) at a flow rate of 2500 ± 200 kg h^−1^. The residence time of the grape mass between the electrodes was 0.09 s. Grape mash was treated with 3.7 square pulses of 100 μs and an electric field strength of 4 kV cm^−1^. Total specific applied energy was 6.2 kJ kg^−1^. Grape mash temperature was measured before and after treatment, and it never increased by more than 2 °C. Control samples consisted of untreated grapes that passed through the PEF treatment chamber with the PEF modulator turned off.

### 2.3. General Analysis of Enological Characteristics

Total acidity, alcohol content, pH, and color intensity were determined according to the methods prescribed by the Organisation Internationale de la Vigne et du Vin [11].

### 2.4. Phenolic Determinations

Total polyphenol index (TPI) and anthocyanins were calculated according to Ribéreau-Gayon [12]; condensed tannins were analyzed according to Sarneckis [13].

### 2.5. High-Performance Liquid Chromatography (HPLC)

The individual polyphenols were analyzed by HPLC according to conditions described by Puértolas [14]. An HPLC Varian ProStar (Varian Inc., Walnut Creek, CA, USA) high-performance liquid chromatography system equipped with a ProStar 240 ternary pump, a ProStar 410 autosampler, and a ProStar 335 photodiode array detector was used. Separation was achieved on a reverse-phase column (LC Luna^®^ 100 Å C18 250 × 4.6 mm; 5 μm particle size, Phenomenex) with a pre-column of the same material (LC Luna 50 × 4.6 mm; 5 μm particle size, Phenomenex). Chromatograms at 280 nm (flavan-3-ols), 320 nm (hydroxycinnamic acids and derivatives), 360 nm (flavonols), and 520 nm (anthocyanins) were recorded. The analyzed phenolic compounds were tentatively identified according to the retention time and the UV-vis spectra of pure standards, and following the UV-vis spectral characteristics published in the literature [1,15,16]. Concentrations of all studied compounds were expressed in mg.L^−1^.

### 2.6. Sensory Analysis

Wines after twelve months of bottle aging, as well as wines after six months of oak aging followed by six months of bottle aging, were sensorially evaluated by seven winemakers (4 men and 3 women ages 40 to 59) belonging to the official panel of “Campo de Borja” Appellation of Origin. Samples of 20 mL of wine at room temperature were presented in clear wine glasses [16] labelled with 3-digit random codes. Panelists were distributed in individual booths, and they were not provided with information regarding the samples to be tested.

The wines were initially evaluated by triangular discriminative analysis using a completely randomized design, associated with a preference test. The objective of this first test was to determine if, after aging in bottle, or in oak barrels plus bottle, the panel could distinguish between the wines obtained from untreated grapes and those obtained from PEF-treated grapes with 3 and 6 days of maceration. After selecting the sample that was considered different, the panelists were also asked to indicate which sample they preferred. The preference analysis result was only taken into consideration when a panelist correctly identified the sample that was different.

Furthermore, a descriptive sensory evaluation of the wines obtained from untreated and PEF-treated grapes after six months of aging in oak barrels plus six months of aging in bottles was conducted. The evaluation protocol was composed of six sensory descriptors, four of which could be affected by the polyphenolic content of the wines: astringency, body, persistence, and color intensity. The magnitude of sensory descriptors was measured on a scale between 0 (very low intensity) and 9 (very high intensity). Results correspond to the average of the scores reported for each panelist.

Panelists expectorate the wine after testing in both the triangle test and the descriptive sensory evaluation.

### 2.7. Statistical Analysis

For wines aged in bottles, three samples from different bottles were analyzed for each condition. In the case of oak aging, samples from two barrels for each condition, and two from subsequent aging in bottles were analyzed.

Data presented in tables and figures represent mean values ± 95% confidence level. Analysis of variance (ANOVA) was carried out using InfoStat statistical software in the 2018 version. The statistical significance of each selected attribute was calculated according to Tukey’s test (*p* ≤ 0.05). The significant difference for triangular tests was determined using statistical tables reported by Roessler, Warren, and Guymon [17].

## 3. Results and Discussion

### 3.1. Physicochemical Analysis of Wine at the Time of Aging in Bottles and Oak Barrels

Table 1 shows the enological characteristics of wines obtained with 3 and 6 days of skin maceration of untreated and PEF-treated grapes. Data correspond to the wines at the time of bottling and aging in oak barrels. Parameter values lay within the range usually observed in *Grenache* variety wines [18,19]. However, the wines obtained in this study had higher alcohol content because the grapes were harvested at the end of the campaign with high sugar concentration.

No statistically significant differences were found in the pH, nor in the total acidity of the four wines, and the differences in ethanol content between the wine with the lowest value and the highest value were less than one unit of ethanol (%v.v^−1^). These differences can be attributed to the varying fermentation processes brought about by the yeast in the separate tanks rather than to the PEF treatment. These results agree to previous studies that showed that PEF does not show any significant effect on wine alcoholic content [20].

For the enological characteristics that depend on phenolic extraction during skin maceration, values increased when grapes were treated with PEF, or when skin maceration was extended from 3 to 6 days. Statistically significant differences were found between the wines obtained from untreated and PEF-treated grapes after 3 or 6 days of skin maceration. Greater differences were found for color intensity (51%) and total anthocyanins (37%) between the wines obtained from untreated and PEF-treated grapes when maceration time was shorter (3 days). The total polyphenol index and the tannin content were 30 to 40% higher for the wines obtained from grapes treated with PEF with the two maceration periods. These effects can be attributed to the electroporation caused by PEF that facilitates the release of intracellular compounds [21].

### 3.2. Evolution of Color Intensity, Anthocyanin Content, Total Phenolic Content, and Tannin Content during Aging in Bottles and Oak Barrels

**Figure 2:**
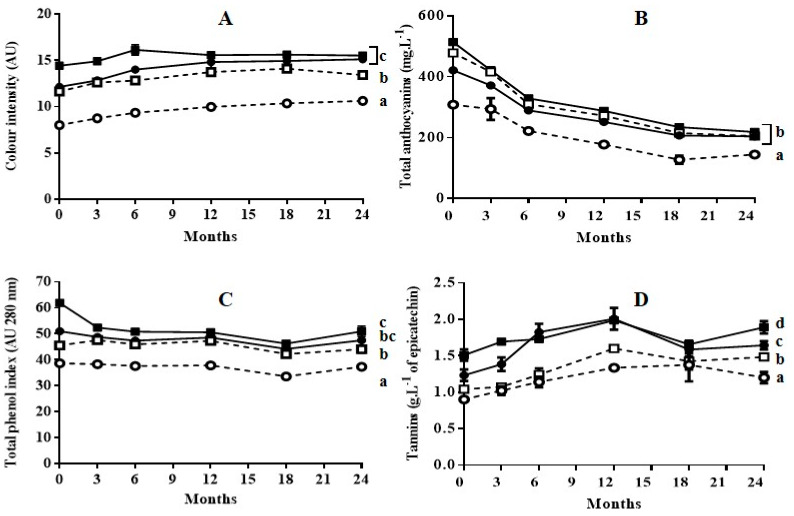
Evolution of color intensity (**A**), total anthocyanins (**B**), total phenol index (**C**), and tannins (**D**) of wines during 24 months of aging in bottles. (○) wine obtained from untreated grapes with 3 days of maceration; (●): wine obtained from PEF-treated grapes with 3 days of maceration; (□): wine obtained from untreated grapes with 6 days of maceration; (■): wine obtained from PEF-treated grapes with 6 day of maceration. Different letters represent significant differences according to one-way ANOVA analysis (*p* < 0.05).

**Figure 3:**
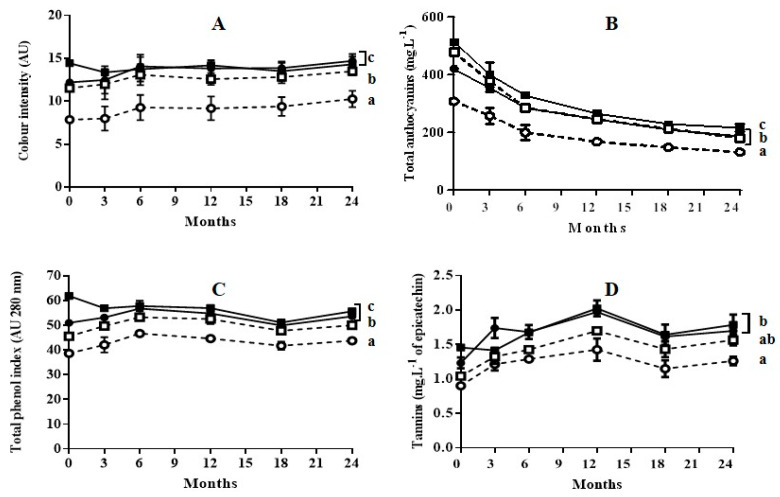
Evolution of color intensity (**A**), total anthocyanins (**B**), total phenol index (**C**), and tannins (**D**) of wines during 6 months of aging in oak barrels plus 18 months of aging in bottles. (○) wine obtained from untreated grapes with 3 days of maceration; (●): wine obtained from PEF-treated grapes with 3 days of maceration; (□): wine obtained from untreated grapes with 6 days of maceration; (■): wine obtained from PEF-treated grapes with 6 day of maceration. Different letters represent significant differences according to one-way ANOVA analysis (*p* < 0.05).

Figure 2 and Figure 3 show the evolution of wine characteristics depending on the polyphenols extracted throughout the maceration stage during aging in bottle for 24 months, and aging in oak barrels for 6 months plus subsequent aging in bottle for 18 months, respectively.

As previously reported during aging of Cabernet Sauvignon wine obtained with grapes treated by PEF [14,22], in general, the application of a PEF treatment prior to the maceration-fermentation stage did not affect the subsequent evolution of color intensity, anthocyanin content, total phenolic content, or tannin content: neither during bottle aging, nor during oak aging followed by bottle aging. In all cases, at the end of the aging process the values for those indexes were lowest for the wine obtained from untreated grapes with 3 days of maceration. It has been reported that wines obtained with techniques such as thermovinification or flash-expansion, which greatly accelerate polyphenol extraction, produce wines that often have poor stability and little structure [23]. This effect has been explained by the fact that these techniques promote the extraction of anthocyanins, but not the extraction of other polyphenols that provide wine structure and anthocyanin stabilization [24]. According to our results, this effect was not observed in the wines obtained with grapes treated by PEF. The evolution of the wine obtained with grapes treated by PEF followed the typical pattern for wine aging reported in the literature [10].

Color intensity values of wines aged in bottles (Figure 2A) or aged in oak barrels plus bottles (Figure 3A) did not change significantly after 24 months of storage. However, aging caused a significant decrease in total anthocyanin content for all four wines (Figure 2B or Figure 3B). In all cases, the reduction in anthocyanin content was more rapid during the first six months of aging. The decrease in anthocyanins during wine aging has been attributed to precipitation and oxidation reactions [25,26,27]. These reactions seem to occur to the same extent in wine obtained from untreated grapes as in wine obtained from grapes treated with PEF. Although anthocyanins are the compounds that mainly account for the red and purple color of wine, the reduction of those compounds during aging did not affect the color intensity. This preservation of color during aging is a consequence of the formation of polymeric pigments lying between anthocyanins and other wine components such as tannins, and of the formation of derived pigments by condensation. Condensation consists in non-covalent links of anthocyanins with colorless molecules or with other anthocyanins [28,29]. Therefore, similarly to the wine obtained from untreated grapes, the wine obtained with PEF-treated grapes contained the molecules that participate in the reactions that are responsible for color stabilization.

The total phenol index (TPI) for the wines aged in bottle obtained with untreated and PEF-treated grapes with 3 days of maceration remained practically constant (Figure 2C). In the case of the wine obtained from PEF-treated grapes with 6 days of maceration, a decrease in TPI was observed after the 3 first months of aging, after which it remained practically constant (Figure 2C). This decrease in TPI could be due to the precipitation of a proportion of polyphenols as a consequence of their high initial concentration at the point of bottling. The evolution of TPI during aging in oak barrels was similar to those in bottle. (Figure 3C). However, for the remainder of the wines, the TPI increased during aging in barrels (6 months), after which it slightly decreased during aging in bottle. Therefore, the extraction of phenolic compounds from the wood responsible for the TPI increment occurred both in wines obtained with untreated grapes as in those with PEF-treated grapes. In the case of the wine obtained with PEF-treated grapes after 6 days of maceration, an increment in TPI was not observed. This was probably because the precipitation of polyphenols exceeded the degree of phenolic extraction from the wood.

Tannins represented in Figure 2D or Figure 3D are formed by the polymerization of the polyphenolic flavan-3-ol monomers catechin and epicatechin [28]. An increment in tannin content up to 12 months of aging was observed as a consequence of the formation of polymer chains with a different degree of polymerization for the four wines aged in bottle or in barrel. After 12 months, this index tended to decrease slowly. Similarly to TPI, no differences in tannin content were observed at the end of aging between the wine obtained from untreated grapes with six days of maceration and the wine obtained from grapes treated by PEF with 3 days of maceration. These results indicate that the concentration of alcohol after 3 days of fermentation was high enough to encourage an elevated rate of extraction of the polyphenols that would form the tannins by polymerization. These compounds, which have a low degree of water solubility, require the presence of ethanol in order to be extracted [30].

### 3.3. Evolution of the Content of Phenolic Families and Individual Phenolics during Aging in Bottles and in Oak Barrels

The concentration of phenolic families (anthocyanins, hydroxycinnamic acids, flavonols, and flavanols) and the individual polyphenols of the four wines after 6, 12, and 24 months of bottle aging, or oak aging followed by bottle aging, are shown in Table 2 and Table 3, respectively.

In the course of the entire 2-year aging period, the total content of phenolic families tended to decrease, independently of PEF treatment or maceration time. Similar results have been observed in the aging of *Cabernet Sauvignon* wine obtained from grapes treated with PEF [14,22].

As in the evolution of the characteristics described above, a higher concentration of individual phenolic compounds was generally observed in the wines obtained from PEF-treated grapes than in those obtained from untreated grapes after an identical maceration period. The differences between the wines obtained from untreated grapes after 6 days of maceration and the wines obtained from PEF-treated grapes with 3 or 6 days of maceration tended to level out in the course of aging, whereby the polyphenolic content of the wines obtained from untreated grapes with 3 days of maceration was always lower. In all cases, no evidence of a particular effect of PEF treatment on the extraction of a specific family or individual phenolic compound was observed.

Monomeric anthocyanins were the predominant polyphenols in all the wines. Among all polyphenolic families, anthocyanins were considerably more reduced in all four wines, either due to reactions associated with the formation of new stable polymeric pigments, or due to degradation reactions. As the color of all four wines remained stable during aging, the loss of monomeric anthocyanins seems to be due to their transformation into more stable pigments in terms of color, rather than to their degradation. Anthocyanin decrease was more pronounced in the wines aging only in bottle than in the wines aging in oak barrels. After 24 months of aging, total individual anthocyanins were 20 to 40% higher for wines aged in barrels.

Malvidin-3-glucoside was the principle anthocyanin, representing practically half of all monomeric anthocyanins in all wines. As in other studies on wine aging, the observed decrease in total monomeric anthocyanins was mainly due to this compound’s notable decrease [14,22]. After 24 months of aging, the concentration of malvidin-3-glucoside decreased significantly in all wines, representing approximately one-third of all monomeric anthocyanins. This decrease was observed in the same proportion in the wines obtained from untreated grapes as in those obtained from PEF-treated grapes. Thus, after the same maceration period, wines obtained with untreated grapes had a lower amount of monomeric anthocyanins compared with the wines obtained from PEF-treated grapes after 24 months in both aging processes.

Glucoside, acetylated, and coumarylated anthocyanins evolved in a similar way, decreasing in the course of aging in wines obtained with untreated and PEF-treated grapes.

A total of three hydrodynamic acids, five flavonols, and two flavanols were identified and quantified in all wines. The evolution of these polyphenolic families in wines obtained from untreated and PEF-treated grapes was similar in the course of aging, either in bottles, or in oak barrels. In all cases, a progressive decrease throughout aging was observed. In general terms, by the end of the aging process, the highest value in these families was observed in the wine obtained from grapes treated with PEF after 6 days of maceration, and the lowest values thereof in the wines obtained after 3 days of maceration with untreated grapes.

Similar results as those discussed regarding different polyphenol families were observed for the individual polyphenols of each family as well. The evolution of individual polyphenols was similar in the two wines obtained from untreated and PEF-treated grapes after aging in bottles, or oak barrels with subsequent bottling. In all cases, a decrease in the concentration of these compounds was observed through time. The wine obtained from grapes treated with PEF after 6 days of maceration presented the highest amount of hydroxycinnamic acids in the course of aging, mainly due to a higher amount of *t*-caftaric acid. This wine also presented the highest amount of flavonols, whereby myricetin-3- glucoside was the most abundant flavonol. In the case of flavanols, after 6 months of aging their content tended to be higher in the wines in oak barrels than in the wines exclusively aged in bottles. This higher content is related to the extraction of flavanols from oak wood [10]. Whereas after 6 months of aging the content of (+)-catechin was higher than the content of (-)-epicatechin in all wines, after 24 months of aging the content of both flavanols was similar.

### 3.4. Sensory Evaluation

Table 4 shows the percentages of correct responses identifying the odd sample in the triangle test and the results of the preference test. Significant sensory differences were detected by the panelists in the wines obtained with untreated or PEF-treated grapes after 3 and 6 days of maceration when aged either in bottles or in oak barrels. All panelists were able to differentiate the wines obtained with untreated or PEF-treated grapes after 3 days of maceration for both types of aging (bottles vs. oak barrels plus bottles). In both cases, a majority of panelists (86%) preferred wines elaborated with PEF-treated grapes. When the wines obtained with untreated and PEF-treated grapes with 6 days of maceration were compared, panelists had more difficulty in differentiating them (71% success) when they had aged in bottles. However, all seven panelists were able to differentiate them when they had aged in barrels. In both cases, panelists likewise preferred the wine obtained from grapes treated with PEF. Finally, independently of the type of aging, panelists were able to differentiate the wines obtained with grapes treated by PEF with 3 days of maceration from the wines obtained with 6 days of maceration with untreated or PEF-treated grapes with a success rate of 86%.

In the preference test, panelists preferred the wines obtained from grapes treated with PEF with longer maceration times when they had aged in bottle (71%) or in oak barrels (57%). Smaller differences were observed in the panelists’ preferences between the wine obtained from grapes treated with PEF and 3 days of maceration (57%) and the wine obtained from untreated grapes and 6 days of maceration (43%), but 71% of the panelists preferred the wine obtained from PEF-treated grapes after six months of aging in oak barrels.

In summary, these results indicate that the improvement in polyphenolic extraction brought about by the application of a PEF treatment prior to maceration permits to obtain wines that are sensorially different from those obtained with untreated grapes. In all cases, panelists preferred wines obtained from grapes treated with PEF after aging in bottles, or in oak barrels. These results support conclusions previously reached in the comparison of physicochemical wine characteristics. The application of a PEF treatment to the grapes permitted to reduce maceration time from 6 to 3 days without negatively affecting the wines’ physicochemical and sensory characteristics. When comparing wines obtained with untreated and PEF-treated grapes after longer maceration periods, smaller differences were observed in characteristics depending on polyphenol extraction, but from a sensory point of view the wine obtained from grapes treated by PEF was preferred by panelists, especially after it had aged 6 months in barrels.

**Figure 4:**
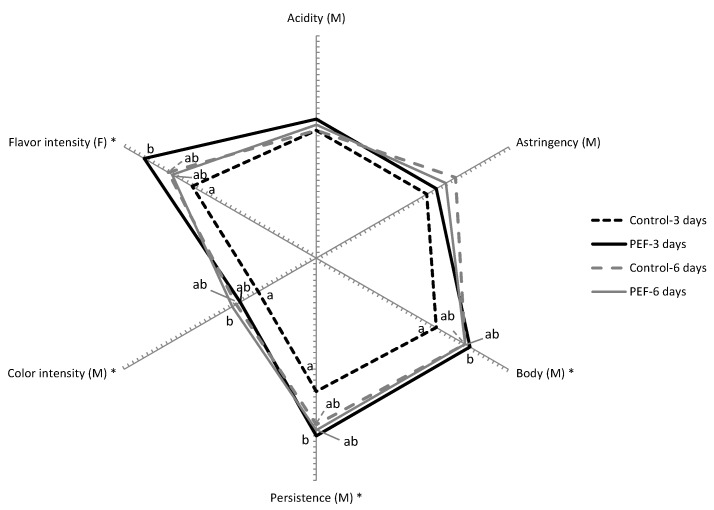
Cobweb diagram of the mean sensory scores (*n* = 7) for the significant mouthfeel (M) and flavor (F) attributes of wines obtained from untreated and PEF-treated grapes with 3 and 6 days of maceration after six months of oak aging and 6 months of bottle aging. Attributes identified with * indicate statistical significance at *p* < 0.05.

Figure 4 displays the sensory profiles of the wines obtained from untreated and PEF-treated grapes with 3 and 6 days of maceration after six months of oak aging and 6 months of bottle aging. This evaluation confirmed the differences among the wines already observed through physicochemical analysis. Wine obtained from untreated grapes and 3 days of maceration was clearly distinct from the remaining wines. It had a lower intensity in flavor, and lower descriptors directly related with polyphenol content such as color intensity, body, astringency, and persistency.

On the other hand, smaller differences in sensory descriptors were obtained between the other three wines, thereby confirming the potential of PEF for the reduction of maceration time without impairing physicochemical characteristics and sensory properties of wine, even after aging.

## 4. Conclusions

Results obtained in this study reveal that the extraction of different families of polyphenols and individual polyphenols was significantly affected by PEF treatments, resulting in wines possessing a higher content of those compounds when compared with wines obtained from untreated grapes after the same amount of maceration days. However, the wine obtained from grapes treated by PEF with different maceration times followed an evolution similar to the wine obtained from untreated grapes in the course of 24 months of bottle aging, or oak aging followed by bottle aging.

Physicochemical and sensory analysis showed that grapes treated by PEF can result in wines not only suitable for everyday consumption, but also in certain high-quality wines that require aging in bottles or in oak barrels Finally, the higher alcohol content of the wines obtained in this study is an issue that should be considered when comparing results obtained in this research with others obtained from PEF-treated grapes with lower concentrations of sugars.

## Figures and Tables

**Table 1:** Physico-chemical characteristics of wines obtained with 3 and 6 days of skin maceration from untreated and PEF-treated grapes at the time of bottling and aging in oak barrels.

		pH	Ethanol (% v.v^−1^)	Titratable Acidity ^a^	CI (AU)	TPI (AU)	Tannin ^b^ Content	Anthocyanins ^c^
**3 days of maceration**	**Untreated**	3.2 ± 0.01a	17.75 ± 0.1a	4.4 ± 0.1a	8.0 ± 0.2a	38.6 ± 0.2a	900.8 ± 12.5a	308.4 ± 11.2a
	**PEF**	3.2 ± 0.03a	17.85 ± 0.1a	4.2 ± 0.2a	12.1 ± 0.1b	51.0 ± 0.3c	1232.7 ± 78.8b	421.1 ± 9.6b
**6 days of maceration**	**Untreated**	3.2 ± 0.01a	17.45 ± 0.1a	4.2 ± 0.1a	11.6 ± 0.1b	45.5 ± 1.4b	1040.7 ± 10.0a	477.9 ± 6.4c
	**PEF**	3.2 ± 0.01a	17.90 ± 0.1a	4.1 ± 0.1a	14.4 ± 0.1c	61.9 ± 0.9d	1457.9 ± 6.8c	513.2 ± 8.0c

Different letters within the same column represent significant differences according to one-way ANOVA analysis (*p* < 0.05); CI color intensity, TPI total polyphenol index, AU absorbance units. ^a^ Expressed as tartaric acid (g L^−1^). ^b^ Expressed as procyanidin (mg L^−1^). ^c^ Expressed as malvidin-3-glucoside (mg L^−1^).

**Table 2:** Evolution of individual phenolic compounds (mg.L^−1^) in wines obtained from untreated and PEF-treated grapes with 3 and 6 days of maceration after 24 months of bottle aging.

	6 Months				12 Months				24 Months			
	3 Days of Maceration		6 Days of Maceration		3 Days of Maceration		6 Days of Maceration		3 Days of Maceration		6 Days of Maceration	
	Control	PEF	Control	PEF	Control	PEF	Control	PEF	Control	PEF	Control	PEF
	Anthocyanins											
delphinidin-3G	6.07 ± 0.35	11.66 ± 3.50	12.58 ± 5.96	15.61 ± 3.35	3.27 ± 0.04	9.62 ± 2.61	0.47 ± 0.05	13.6 ± 0.91	3.75 ± 0.25	8.64 ± 2.83	11.73 ± 0.18	10.01 ± 0.21
cyanidin-3G	1.63 ± 0.50	8.50 ± 1.98	3.27 ± 0.78	3.41 ± 0.72	3.18 ± 1.41	5.39 ± 0.11	9.43 ± 1.41	3.92 ± 2.86	0.76 ± 0.54	0.94 ± 0.58	1.21 ± 0.45	0.78 ± 0.65
petunidin-3G	12.02 ± 1.23	16.14 ± 2.19	19.18 ± 3.39	20.61 ± 0.19	2.51 ± 0.43	9.05 ± 0.18	10.33 ± 7.86	10.18 ± 1.99	1.99 ± 0.66	2.91 ± 0.73	1.29 ± 1.01	1.55 ± 0.24
peonidin-3G	8.67 ± 0.07	13.92 ± 1.45	15.19 ± 4.62	17.54 ± 0.90	5.02 ± 0.80	10.98 ± 0.96	5.97 ± 0.81	17.03 ± 2.15	3.39 ± 0.99	7.98 ± 0.01	6.79 ± 0.04	10.29 ± 2.02
malvidin-3G	104.87 ± 0.50	136.18 ± 7.43	134.58 ± 15.89	185.13 ± 6.71	32.47 ± 1.43	50.25 ± 2.63	60.04 ± 10.04	68.07 ± 10.84	9.63 ± 0.36	26.11 ± 0.76	29.56 ± 3.22	34.27 ± 4.53
delphinidin-3G-Ac	9.23 ± 0.52	13.51 ± 2.14	20.75 ± 4.49	14.53 ± 1.80	4.65 ± 0.06	9.56 ± 4.82	0.10 ± 0.07	11.07 ± 1.04	2.10 ± 1.05	3.12 ± 0.25	1.48 ± 1.83	4.19 ± 0.90
cyanidin-3G-Ac	1.32 ± 0.58	2.36 ± 0.28	2.43 ± 0.42	3.02 ± 0.41	3.71 ± 0.24	2.87 ± 1.99	14.11 ± 0.94	2.21 ± 1.06	1.01 ± 0.35	0.98 ± 0.78	1.20 ± 0.48	0.72 ± 0.25
petunidin-3G-Ac	3.25 ± 1.08	10.35 ± 0.25	13.48 ± 6.01	7.74 ± 1.93	6.89 ± 0.61	12.61 ± 4.79	8.74 ± 1.91	11.31 ± 0.24	1.05 ± 0.61	1.87 ± 1.41	3.52 ± 0.02	2.01 ± 1.05
malvidin-3G-Ac + peonidin-3G-Ac	7.35 ± 1.55	26.33 ± 3.39	23.95 ± 0.01	30.62 ± 3.04	10.28 ± 0.88	18.33 ± 0.34	17.19 ± 6.70	27.00 ± 1.07	4.50 ± 0.01	9.05 ± 1.48	11.45 ± 4.01	11.8 ± 2.56
delphinidin-3G-Cm	1.42 ± 1.72	9.17 ± 1.32	10.51 ± 1.09	12.64 ± 2.52	3.44 ± 0.64	8.07 ± 3.90	8.58 ± 0.42	7.59 ± 5.26	1.11 ± 0.17	1.67 ± 0.30	1.15 ± 0.47	1.07 ± 0.35
cyanidin-3G-Cm	0.85 ± 0.54	1.08 ± 0.43	1.35 ± 0.98	1.65 ± 0.11	1.07 ± 0.41	1.88 ± 0.05	2.03 ± 0.36	2.48 ± 0.68	0.78 ± 0.18	1.10 ± 0.06	1.39 ± 0.08	1.17 ± 0.24
petunidin-3G-Cm	2.01 ± 0.77	7.71 ± 5.63	11.44 ± 0.15	6.49 ± 1.11	2.97 ± 0.50	2.48 ± 2.58	3.64 ± 0.66	3.17 ± 2.34	0.66 ± 0.03	0.75 ± 0.27	0.69 ± 0.03	2.26 ± 2.42
peonidin-3G-Cm	4.14 ± 2.08	3.96 ± 1.71	8.99 ± 7.73	8.09 ± 0.07	5.43 ± 0.73	7.68 ± 0.94	5.61 ± 0.03	5.16 ± 1.52	1.42 ± 0.09	2.02 ± 0.01	3.29 ± 0.11	2.56 ± 0.48
malvidin-3G-Cm	7.94 ± 4.70	7.72 ± 3.42	14.27 ± 2.7	14.19 ± 1.57	4.69 ± 1.42	6.29 ± 1.95	8.72 ± 1.79	7.86 ± 0.63	1.54 ± 0.18	3.46 ± 0.47	4.93 ± 0.48	7.28 ± 1.43
**Total individual anthocyanins**	**170.78 a**	**268.61 b**	**291.96 bc**	**341.26 c**	**89.56 a**	**155.06 b**	**154.96 b**	**190.63 c**	**33.66 a**	**70.59 b**	**79.67 bc**	**89.93 c**
	Hydroxycinnamic acids											
*t*-caftaric acid	8.70 ± 0.99	16.6 ± 2.55	13.05 ± 1.48	23.90 ± 1.98	7.85 ± 0.07	13.50 ± 0.57	14.30 ± 0.28	16.95 ± 1.06	2.80 ± 0.14	8.40 ± 0.28	8.50 ± 0.85	8.45 ± 0.92
*p*-coumaric acid	2.90 ± 0.28	2.95 ± 1.06	3.90 ± 0.42	4.45 ± 0.21	1.65 ± 0.35	2.70 ± 0.14	2.55 ± 0.35	3.35 ± 0.49	0.30 ± 0.01	1.65 ± 0.64	2.60 ± 0.14	3.70 ± 0.14
caffeic acid	0.50 ± 0.01	1.55 ± 0.35	0.70 ± 0.42	1.40 ± 0.28	0.30 ± 0.01	0.50 ± 0.14	0.40 ± 0.28	1.30 ± 0.01	1.05 ± 0.07	1.05 ± 0.21	0.65 ± 0.07	1.45 ± 0.07
**Total ind. hydrocynnamic ac**	**12.10 a**	**21.10 ab**	**17.65 a**	**29.75 b**	**9.80 a**	**16.70 b**	**17.25 b**	**21.6 c**	**4.15 a**	**11.10 b**	**11.75 bc**	**13.60 c**
	Flavonols											
myricetin-3G	5.65 ± 0.07	8.35 ± 1.91	7.50 ± 0.14	9.15 ± 1.20	2.40 ± 0.57	6.01 ± 0.28	5.25 ± 0.35	6.95 ± 1.06	0.50 ± 0.01	4.60 ± 0.57	3.15 ± 0.35	4.25 ± 1.06
myricetin	0.45 ± 0.21	3.05 ± 0.21	0.55 ± 0.07	2.65 ± 0.78	0.20 ± 0.01	1.05 ± 0.21	0.45 ± 0.07	1.60 ± 0.01	0.10 ± 0.01	0.20 ± 0.14	0.40 ± 0.14	0.40 ± 0.14
isorhamnetin-3O-glucoside	0.30 ± 0.14	1.05 ± 0.07	1.40 ± 0.28	1.30 ± 0.71	0.40 ± 0.57	0.15 ± 0.21	0.60 ± 0.14	3.30 ± 0.14	nd	0.15 ± 0.21	0.40 ± 0.01	0.15 ± 0.07
quercetin-3G	4.60 ± 1.98	7.30 ± 0.42	7.75 ± 1.34	8.90 ± 0.99	2.05 ± 0.49	5.2 ± 0.42	5.50 ± 0.14	6.40 ± 1.27	2.60 ± 0.42	5.30 ± 0.28	5.90 ± 0.28	7.25 ± 2.33
quercetin	1.30 ± 0.57	1.35 ± 0.07	0.70 ± 0.28	1.01 ± 0.42	0.70 ± 0.14	0.65 ± 0.07	0.65 ± 0.07	0.55 ± 0.07	0.35 ± 0.07	0.40 ± 0.14	0.30 ± 0.28	0.70 ± 0.28
**Total individual flavonols**	**12.30 a**	**21.10 b**	**17.90 ab**	**23.00 b**	**5.75 a**	**13.05 b**	**12.45 b**	**18.80 c**	**3.55 a**	**10.65 b**	**10.15 ab**	**12.75 b**
	Flavanols											
(+)-catechin	10.15 ± 0.07	18.05 ± 0.92	17.35 ± 1.91	20.75 ± 2.19	7.05 ± 2.76	7.20 ± 1.27	11.50 ± 0.71	13.01 ± 1.41	6.20 ± 0.57	6.95 ± 1.48	7.85 ± 0.92	10.10 ± 1.27
(-)-epicatechin	7.25 ± 0.49	13.55 ± 2.33	14.80 ± 1.27	15.20 ± 1.13	10.95 ± 1.48	14.5 ± 0.71	13.50 ± 0.71	17.50 ± 2.12	5.45 ± 1.20	8.00 ± 1.13	10.25 ± 1.06	10.55 ± 2.05
**Total individual flavanols**	**17.40 a**	**31.60 b**	**32.15 b**	**35.95 b**	**18.00 a**	**21.70 ab**	**25.00 bc**	**30.51 c**	**11.65 a**	**14.95 a**	**18.10 a**	**20.65 a**

^a^ nd: not detected. G: glucoside. Ac: acylated. Cm: coumarylated.

**Table 3:** Evolution of individual phenolic compounds (mg L^−1^) in wines obtained from untreated and PEF-treated grapes with 3 and 6 days of maceration after 6 months of aging in oak barrels plus 18 months bottle aging.

	6 Months				12 Months				24 Months			
	3 Days of Maceration		6 Days of Maceration		3 Days of Maceration		6 Days of Maceration		3 Days of Maceration		6 Days of Maceration	
	Control	PEF	Control	PEF	Control	PEF	Control	PEF	Control	PEF	Control	PEF
	Anthocyanins											
delphinidin-3G	7.14 ± 0.06	12.13 ± 1.91	9.44 ± 0.98	11.57 ± 2.11	5.64 ± 0.74	8.19 ± 0.63	14.80 ± 0.03	16.33 ± 1.46	4.07 ± 0.24	10.67 ± 1.1	11.85 ± 0.1	11.17 ± 0.2
cyanidin-3G	1.49 ± 0.86	6.31 ± 3.88	4.41 ± 0.13	3.56 ± 0.61	3.30 ± 1.69	2.80 ± 0.38	5.61 ± 0.52	3.76 ± 1.37	1.61 ± 0.01	3.53 ± 2.1	3.13 ± 0.12	3.12 ± 1.5
petunidin-3G	13.79 ± 0.30	16.41 ± 1.28	14.29 ± 2.51	18.42 ± 1.80	6.46 ± 0.79	14.24 ± 1.50	7.76 ± 1.99	11.31 ± 0.60	0.72 ± 0.01	3.11 ± 0.1	4.77 ± 1.2	7.87 ± 1.4
peonidin-3G	8.03 ± 2.04	13.69 ± 3.54	23.74 ± 6.39	15.52 ± 3.09	8.21 ± 1.74	10.64 ± 3.93	11.54 ± 0.23	19.74 ± 0.18	10.65 ± 1.07	8.54 ± 0.6	8.98 ± 0.8	9.90 ± 1.8
malvidin-3G	75.39 ± 4.80	144.36 ± 12.05	127.93 ± 19.59	182.36 ± 12.39	25.2 ± 1.77	52.24 ± 2.90	50.59 ± 2.61	59.18 ± 1.78	12.68 ± 3.01	23.27 ± 2.6	31.51 ± 3.8	40.63 ± 6.7
delphinidin-3G-Ac	8.46 ± 0.49	10.85 ± 4.07	15.87 ± 1.51	21.21 ± 0.45	2.64 ± 0.24	7.68 ± 0.09	9.26 ± 0.58	11.56 ± 5.75	1.33 ± 0.03	4.69 ± 1.31	10.22 ± 0.1	7.61 ± 0.8
cyanidin-3G-Ac	2.86 ± 0.58	3.36 ± 0.34	2.80 ± 0.06	3.71 ± 0.22	0.62 ± 0.18	1.14 ± 0.12	1.34 ± 0.09	2.82 ± 2.03	0.59 ± 0.36	0.02 ± 0.04	2.29 ± 0.10	2.45 ± 0.05
petunidin-3G-Ac	2.87 ± 0.23	7.85 ± 2.81	7.97 ± 0.88	13.68 ± 2.79	3.50 ± 1.86	9.42 ± 2.41	7.96 ± 2.09	13.44 ± 2.77	1.82 ± 0.14	2.45 ± 1.2	2.58 ± 1.10	3.00 ± 0.50
malvidin-3G-Ac+peonidin-3G-Ac	13.38 ± 1.16	34.85 ± 7.99	21.14 ± 0.78	36.58 ± 2.54	10.67 ± 2.48	16.96 ± 3.01	22.01 ± 0.27	20.09 ± 5.04	5.73 ± 0.59	10.93 ± 1.4	16.77 ± 1.4	17.38 ± 5.2
delphinidin-3G-Cm	2.68 ± 1.21	5.10 ± 0.23	8.78 ± 3.46	10.09 ± 2.01	2.52 ± 0.53	5.28 ± 0.80	5.43 ± 0.88	3.98 ± 0.19	1.10 ± 0.18	4.14 ± 0.35	2.17 ± 0.23	3.73 ± 0.07
cyanidin-3G-Cm	1.11 ± 0.04	1.96 ± 0.22	3.03 ± 1.44	1.29 ± 2.65	0.57 ± 0.02	1.88 ± 0.72	1.46 ± 0.34	2.44 ± 0.05	1.04 ± 0.42	2.50 ± 0.02	1.34 ± 0.09	0.55 ± 0.20
petunidin-3G-Cm	1.75 ± 0.65	6.56 ± 3.70	7.22 ± 0.95	8.45 ± 0.01	1.15 ± 0.70	3.01 ± 0.97	4.13 ± 2.30	3.82 ± 1.97	1.55 ± 0.39	2.30 ± 0.91	1.81 ± 0.29	1.12 ± 0.23
peonidin-3G-Cm	2.99 ± 0.87	14.58 ± 0.37	4.38 ± 1.64	12.71 ± 1.41	1.42 ± 0.35	4.74 ± 0.41	7.62 ± 5.27	5.36 ± 0.33	1.81 ± 0.73	3.29 ± 0.38	1.37 ± 0.52	5.60 ± 0.04
malvidin-3G-Cm	5.09 ± 0.89	7.43 ± 0.73	19.32 ± 0.01	12.72 ± 1.43	3.86 ± 1.09	8.60 ± 2.43	5.23 ± 2.71	9.12 ± 1.53	1.89 ± 0.26	5.03 ± 0.75	5.28 ± 0.10	10.11 ± 0.5
**Total individual anthocyanins**	**147.04 a**	**285.43 b**	**270.32 b**	**351.88 c**	**75.74 a**	**146.80 b**	**154.74 b**	**182.96 c**	**46.60 a**	**84.48 b**	**104.07 bc**	**124.23 c**
	Hydroxycinnamic acids											
*t*-caftaric acid	9.90 ± 1.84	16.80 ± 2.12	13.85 ± 0.21	23.60 ± 4.53	7.40 ± 1.41	14.55 ± 1.20	13.60 ± 0.14	17.50 ± 0.85	3.60 ± 0.85	8.60 ± 0.85	9.10 ± 0.28	10.70 ± 0.2
*p*-coumaric acid	2.65 ± 0.49	3.20 ± 0.57	3.35 ± 0.07	3.60 ± 0.28	2.20 ± 0.14	2.75 ± 0.35	3.10 ± 0.28	3.10 ± 0.57	0.20 ± 0.14	1.60 ± 0.01	1.90 ± 1.84	1.50 ± 0.57
caffeic acid	0.55 ± 0.07	1.25 ± 0.92	0.50 ± 0.14	0.65 ± 0.21	0.50 ± 0.02	0.60 ± 0.02	0.55 ± 0.07	1.40 ± 0.01	0.75 ± 0.07	1.25 ± 0.07	0.75 ± 0.07	1.50 ± 0.42
**Total ind. hydrocynnamic ac**	**13.10 a**	**21.25 ab**	**17.70 ab**	**27.85 b**	**10.10 a**	**17.90 b**	**17.25 b**	**22.00 b**	**4.55 a**	**11.45 b**	**11.75 b**	**13.70 b**
	Flavonols											
myricetin-3G	4.90 ± 0.28	7.75 ± 0.92	7.40 ± 0.57	11.45 ± 1.63	2.60 ± 0.71	6.80 ± 0.85	5.35 ± 0.35	7.01 ± 1.13	0.85 ± 0.21	5.05 ± 0.35	3.25 ± 0.35	4.95 ± 0.07
myricetin	0.35 ± 0.35	2.65 ± 0.78	0.90 ± 0.01	2.70 ± 0.99	0.20 ± 0.14	1.05 ± 0.07	0.45 ± 0.07	1.60 ± 0.14	nd	0.30 ± 0.14	0.45 ± 0.07	0.90 ± 0.14
isorhamnetin-3O-glucoside	1.15 ± 0.64	2.75 ± 0.07	0.95 ± 0.92	3.85 ± 2.47	0.15 ± 0.21	0.20 ± 0.28	0.70 ± 0.42	0.20 ± 0.01	0.15 ± 0.21	0.20 ± 0.28	0.45 ± 0.07	0.20 ± 0.01
quercetin-3G	3.10 ± 0.42	7.35 ± 2.47	6.90 ± 0.85	8.45 ± 0.92	2.45 ± 0.21	5.45 ± 0.64	5.75 ± 0.07	6.85 ± 0.92	2.55 ± 0.35	4.35 ± 0.21	5.65 ± 0.78	7.55 ± 0.92
quercetin	0.75 ± 0.64	0.05 ± 0.07	1.01 ± 0.14	1.35 ± 0.64	0.60 ± 0.14	0.35 ± 0.35	0.75 ± 0.07	0.75 ± 0.35	nd	0.20 ± 0.28	0.32 ± 0.14	0.75 ± 0.35
**Total individual flavonols**	**10.25 a**	**20.55 b**	**17.15 ab**	**27.80 b**	**6.00 a**	**13.85 b**	**13.00 b**	**16.40 b**	**3.50 a**	**10.10 b**	**10.10 b**	**14.35 c**
	Flavanols											
(+)-catechin	12.55 ± 0.64	22.3 ± 1.84	19.85 ± 0.21	29.01 ± 0.99	10.01 ± 0.01	8.45 ± 0.49	8.50 ± 3.54	10.75 ± 3.18	4.45 ± 2.05	9.05 ± 0.49	11.00 ± 1.2	8.90 ± 1.56
(-)-epicatechin	8.90 ± 2.12	19.25 ± 2.62	14.55 ± 1.06	18.1 ± 1.84	5.85 ± 0.07	23.5 ± 0.71	17.01 ± 2.83	24.5 ± 4.95	5.55 ± 0.78	10.50 ± 0.7	10.50 ± 0.7	13.5 ± 3.54
**Total individual flavanols**	**21.45 a**	**41.55 bc**	**34.40 b**	**47.10 c**	**15.85 a**	**31.95 c**	**25.50 b**	**35.25 c**	**10.00 a**	**19.55 a**	**21.50 a**	**22.40 a**

^a^ Nd: not detected. G: glucoside. Ac: acylated. Cm: coumarylated.

**Table 4:** Triangle test and percentage of preference for each of the comparisons among wines obtained from untreated and PEF-treated grapes with 3 and 6 days of maceration after aging in bottles (12 months), and in oak barrels (6 months) followed by bottle aging (6 months).

		Triangle Test (Percentage of Correct Responses) ^a^	Preference Test (Percentage of Preference) ^b^			
			Control-3 Days	PEF-3 Days	Control-6 Days	PEF-6 Days
**Bottles**	**Untreated-3 days/PEF-3 days**	100 ***	14	86	-	-
	**Untreated-6 days/PEF-6 days**	71 *	-	-	29	71
	**PEF-3 days/PEF-6 days**	86 **	-	29	-	71
	**PEF-3 days/Untreated-6 days**	86 **	-	57	43	-
**Oak barrels**	**Untreated-3 days/PEF-3 days**	100 ***	14	86	-	-
	**Untreated-6 days/PEF-6 days**	100 ***	-	-	43	57
	**PEF-3 days/PEF-6 days**	86 **	-	14	-	86
	**PEF-3 days/Untreated-6 days**	86 **	-	71	29	-

^a^ Significant differences between the samples *, **, *** are statistically significant at *p* ≤ 0.05, *p* ≤ 0.01 and *p* ≤ 0.001 respectively. ^b^ Proportion of preferences statistically different to 50 % according to chi-square test.
